# The role of BaTiO_3_ nanoparticles as photocatalysts in the synthesis and characterization of novel fruit dyes is investigated

**DOI:** 10.1002/jemt.24678

**Published:** 2024-10-27

**Authors:** N. N. Shafeera, D. Saravanakkumar, K. Mohamed Rafi, A. Ayeshamariam, K. Kaviyarasu

**Affiliations:** ^1^ Department of Physics Khadir Mohideen College, (Affiliated to Bharathidasan University, Tiruchirappalli) India; ^2^ PG and Research Department of Physics Thiagarajar College Madurai Tamil Nadu India; ^3^ Department of Botany Jamal Mohamed College (Autonomous) Affiliated to Bharathidasan University Tiruchirappalli Tamil Nadu India; ^4^ UNESCO‐UNISA Africa Chair in Nanosciences/Nanotechnology Laboratories, College of Graduate Studies University of South Africa (UNISA) Pretoria South Africa

**Keywords:** BaTiO_3_ nanoflakes, electron microscopy, phase transition, photocatalytic activity, phytochemical extract, *p*‐type conductivity

## Abstract

In the present work, the photocatalytic activity against the natural dye extracted from the novel fruits has been studied by the BaTiO_3_ nanoparticles (NPs) under a ultra‐violet (UV) light source. The large concentrations of an essential phenolic agent present in this phytochemical extract superimposed with cloths fibers make strong stain and degrade into another form of toxic, which is excluded from the many textiles industries as the colorful waste waters without recycling and removal of that dye pigments have been investigated using both photodegradation and photoluminescence techniques. The entitled nanoparticles (NPs) were prepared using the soft chemical root‐modified solvothermal synthesis combo method and exposure to heat treatment such that the annealing process has been done for different temperatures ranging from 100°C to 250°C. As for as concern the characterization, as a start, structural and morphology studies have been reported here that highly crystalline oriented peaks data using powder x‐ray diffraction techniques (PXRD) as well as the surface morphology including the size, shape, and mass distribution using the field emission scanning electron microscopy (FESEM) techniques, which purely belong to rutile tetragonal structure of the crystal system and circular and noncircular flakes like rough surface morphology materials respectively. The lattice dissociation constant ‘*ε*’ value of the BaTiO_3_ NPs has determined to be ~2.71 × 10^−3^ using the Williamson–Hall (W–H plot) analysis of crystallographic data. In the UV visible spectroscopy findings, since the extreme quantum confinement of BaTiO_3_ nanoflakes/nanodisc, the optical energy bandgap has been estimated to be a range of 1.98 to 2.67 eV (~2.48 eV) found from the *Tauc plot* analysis, which contributes to the significantly owing to the enhanced photocatalytic efficiency with excellent performance along exciton formation, superoxide ions, and hydroxyl free radicals generations under UV–vis light irradiation resulting in efficient degradation of typical novel fruit organic dye. Photoluminescence spectra observed at room temperature and low temperature have been observed for the BaTiO_3_ nanoflakes, which exhibit the blue emission due to the crystalline defects such as the appearance of Ba vacancies leads to the conceivable beginning of *p*‐type conductivity and the origination of free exciton emission reveals the direct bandgap transition nature of nanoflakes.

**Research Highlights:**

- According to our findings, 89.71% of the natural syzygium cumin is degraded by photocatalysis reaction.
- As a plausible mechanism for the destruction of natural dyes under solar light, photocatalytic destruction has been proposed.
- The reaction between these reactive free radical species leads to high efficiency photodegradation with a short decay time.
- In addition to water treatment and environmental cleaning applications, the excellent performance of this photocatalyst makes it a promising candidate for other applications.
- Hence, the synthesized BaTiO_3_ nanoflakes showcase a highly significant advancement towards the development of a textiles dye recycling method.

## Graphical abstract

One of the fundamental driving forces in the discovery of novel nanomaterials is that between 89.71% of the natural syzygium cumin is degraded by photocatalysis. BaTiO_3_ nanocomposite exhibits enhanced photocatalytic activity when the temperature is varied to reduce electron–hole reunion. It has been proposed that photocatalytic destruction is a plausible mechanism for destroying natural dyes in the presence of solar light. A short decay time and high photodegradation efficiency are the results of the reactions between these free radical species. Photocatalysts are excellent candidates for other applications, including water treatment and environmental cleaning.

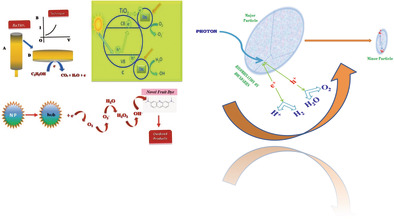

## 1 INTRODUCTION

For the few decades, the great attention given by the BiTiO_3_ nanomaterials, which were reported to belong to the complex oxide nanocomposite family performing in the digital electronics factories since they have an accurate response on nonlinear ferroelectric (NLFE), photostability, and good relative permittivity (*ε*). Generally, semiconductor oxide materials like BiTiO_3_ show great reports among the research people for the dye and unwanted contaminants existing in wastewater in the purification process in many countries facing water pollution problems and alternative methods of useable water at hydro plants under recycling toward people's needs such as basic volume of water at home and many textile industries. Under basic support of UV irradiation arrangement macro composition of any dye is split up into micro compositions using the BiTiO_3_ by following the way of photodegradation mechanism in which strong free radicals emerge due to the appropriate oxidation process in resulting be simple composition products as for as concern the surface adsorbents of oxygen delivery to execute the photodegradation mechanism (Alex et al., 2019). Many preparation methods opt for the cubic perovskite crystal structured BaTiO_3_ nanoparticles (NPs) formations such as coprecipitation, microwave‐assisted solution, soft chemical root, sol–gel, modified perfume spray pyrolysis, hydrothermal, sol–gel combustion, conventional and modified hydrothermal with surfactant assistant and pressure, spray pyrolysis and silar. Many geometrical shapes such as nanoflakes, nanodisc, nanoball, nanorods, nanowires, nanobeads, and nanocrystals with the higher surface‐to‐volume (s/v) ratio cubic and hexagonal wurtzite structures are responsible for releasing the required both positive and negative free ionic species under oxidation and reduction process mechanism in addition with the occurrence of electron–hole (e^−^/h^+^) pair formation due to a large number of ions vacancies present in the regular and periodic arrangement of atoms in the crystalline phase for the photocatalytic and inhibiting the microbes in a standard as well as efficient way (Nageri & Kumar, 2018). Apart from the many crystalline structures, cubic BaTiO_3_ NPs with lattice defects and distortion at RT support the catalytic property in presence of an ultraviolet ray and trigger the ions formations for the same mechanisms (Suematsu et al., 2018).

In the series semiconductors and optoelectronics promising nanomaterials, BaTiO_3_ NPs are the multidisciplinary promising candidates in the semiconductor age. Barium titanate (BaTiO_3_) is one of the most explored electronic ceramics with efficient perovskite ferroelectric properties with unique crystalline nature and with high dielectrics constant since there is occurrence of shift of oxygen and titanium leads to sustaining the dipole moments at RT. There are very useful in the optical storage with high density, dye degradation technology, dielectric amplification sensor, NLO fibers and optical device, pulsed laser–mirror device, multilayer ceramic capacitors, electro optic devices, thermistors, photocatalytic water treatment, and antimicrobial with biofilm drug medicinal field, OFC industry (Cui et al., 2020; Kappadan & Gebreab, 2016; Wang et al., 2021).

## 2 EXPERIMENTAL PROCEDURE

There was no further purification of any of the precursor chemicals and reagents used in this study. Precipitation of the precursor solution is generally effective with sodium hydroxide at optimum pH conditions. Hence, titanium oxide and barium chloride with different % of m wt were dissolved in 60 mL deionized water with enough sodium hydroxide (NaOH) solution to maintain a pH value at 8 since according to the review, this is the appropriate pH value. The above solution was stirred continuously for 24 hrs at 80°C with the addition of 0.5 g of each of the modifiers with mass ratio of 1:1 sodium hydroxide (NaCl) and ethylenediaminetetraacetic acid (EDTA). A 2 hrs autoclave treatment at 150°C was conducted after the solution was carefully shifted into a viscous sol. As soon as the heating system is turned off, the precipitate is added to a solution of ethanol to cool down, and the well settled precipitates (BaTiO_3_) are centrifuged at 5000 rpm, washed with ethanol and deionized water five times for well purification, and then dried in air at 60°C for 12 hrs.

### 2.1 Characterization studies

In this present work, BaTiO_3_ NPs have been prepared using a soft chemical root‐modified solvothermal synthesis combo method and then annealed with different temperature. Using solar illumination, we investigated the efficiency of quantum dots for photocatalytic movement. BaTiO_3_ composites were characterized by S‐4800 Hitachi field emission scanning electron microscopy (FESEM) for their morphology, microstructure, and elemental analyses. The composite has remarkable reusability for catalysis and continuous photodegradation of syzygium cumini fruit dye. As a reference, BaSO_4_ powder was used for ultraviolet–visible diffuse reflectance spectroscopy (UV–vis DRS) recording on a Varian Cary 5000 UV–vis spectrophotometer. Fluorescence emission spectra were obtained from a 150 W xenon lamp using a Hitachi F‐4600 fluorescence system (Japan). A fluorescence spectrophotometer (FLSP‐920, Edinburgh Instruments) was used to collect time‐resolved fluorescence decay spectra were reported in detail.

## 3 RESULTS AND DISCUSSION

### 3.1 PXRD analysis

**FIGURE 1:**
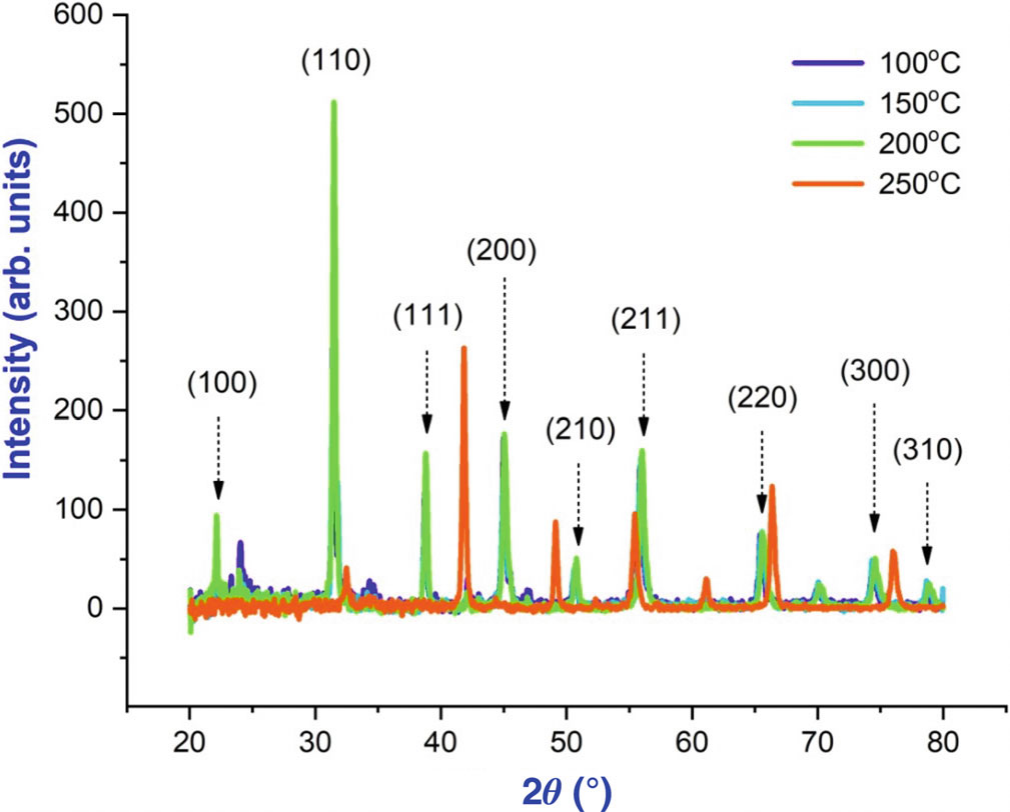
XRD pattern of BaTiO_3_ nanoparticle at various temperatures.

**FIGURE 2:**
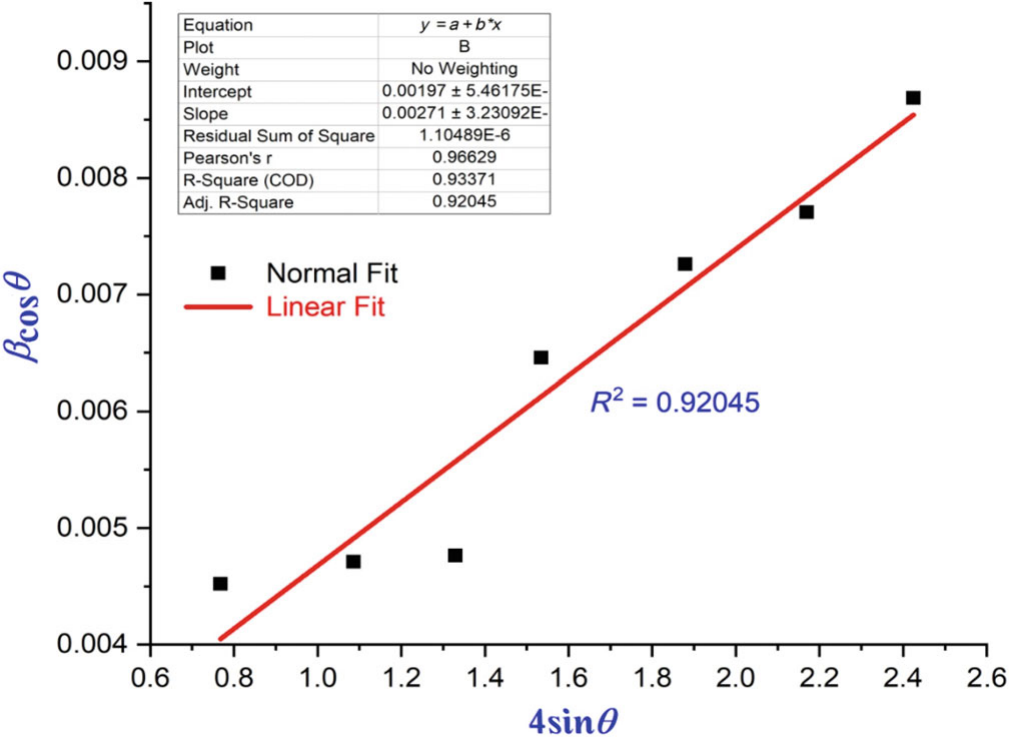
Williamson–Hall plot of BaTiO_3_ nanoparticle at 200°C.

Investigation of the crystal structure of the synthesized NPs prepared by sonochemical method structural studies was analyzed by using x‐ray diffraction (XRD) diffractometer. BaTiO_3_ synthesized shows clear reflections absorbed that 25°, 37°, 47°, 53°, and 62° along with miller indices values of the diffraction planes of (112), (002), (211), (310), and (222). As a result of the preparation process, the samples exhibit well‐defined peaks, indicating a high degree of crystallinity (Amaechi et al., 2019; Ray et al., 2021). Easily indexed with pure hexagonal phase were all the diffraction peaks. At 500°C, the average crystallite size of the sample was calculated using Scherer's formula (*D* = k*λ*/*β*cos*θ*) as 28 nm. In the analysis, the values of lattice constants obtained were *a* = *b* = 10.405 Å, c = 6.4388 Å and *a* = *b* = 10.2499 Å, *c* = 6.3805 Å. Volume of a unit cell (V) is 1804.7671 Å^3^. The *c*‐axis is known to be the preferred direction of growth for apatite crystals. Figure 1 shows XRD patterns for nanoparticles (NPs) of BaTiO_3_ in different concentrations (Wu et al 2019). According to, annealing slightly shifts peak diffraction angle towards Williamson–Hall (W‐H) plot of BaTiO_3_ NP have higher values as shown in Figure 2 (Amaechi et al., 2019).

### 3.2 UV–vis spectra analysis

**FIGURE 3:**
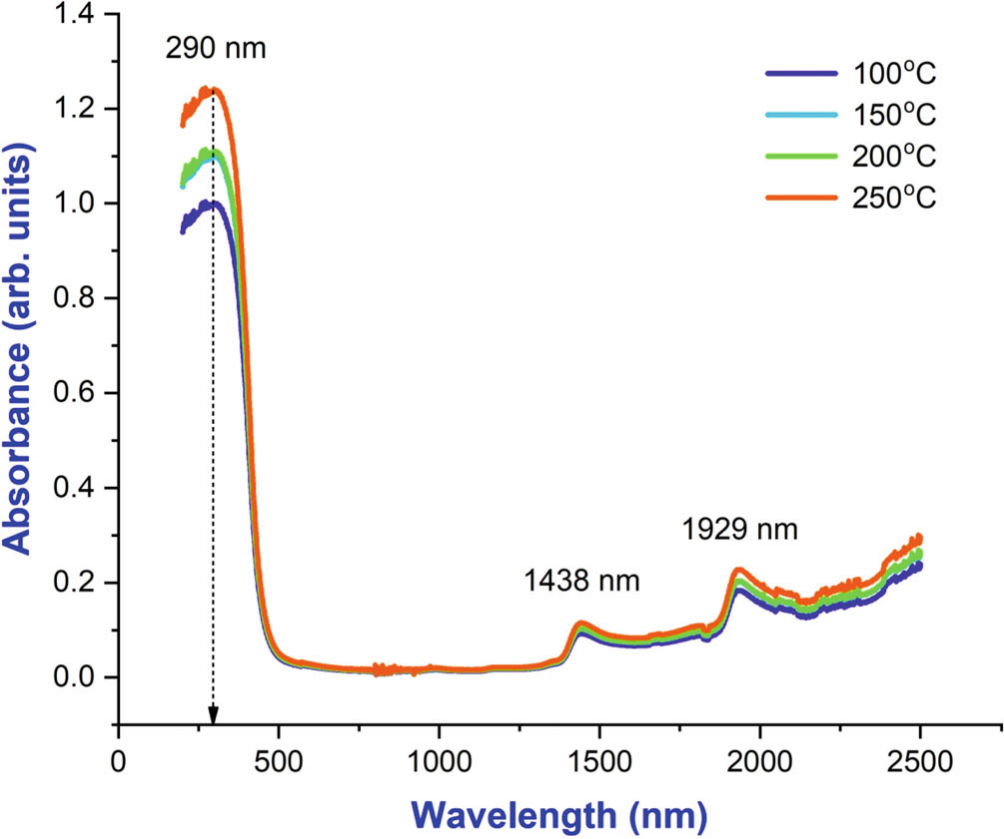
UV–vis absorbance spectrum of BaTiO_3_ nanoparticle at various temperatures.

**FIGURE 4:**
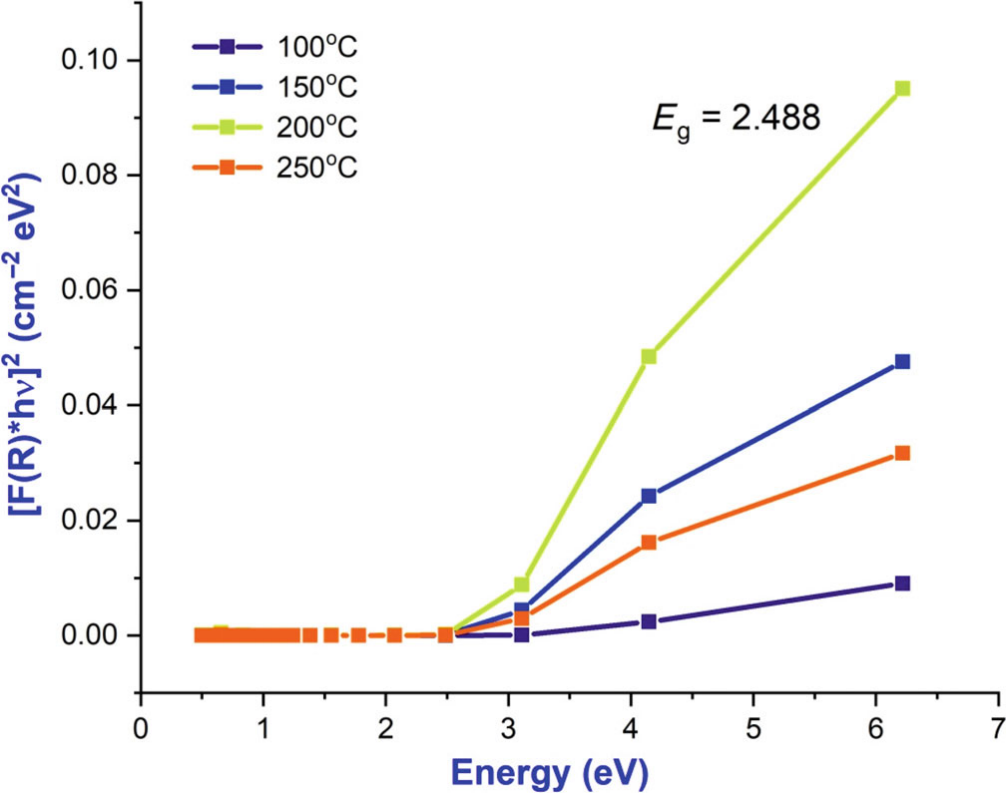
Energy bandgap of annealed BaTiO_3_ nanoparticle at 100°C, 150°C, 200°C and 250°C.

**FIGURE 5:**
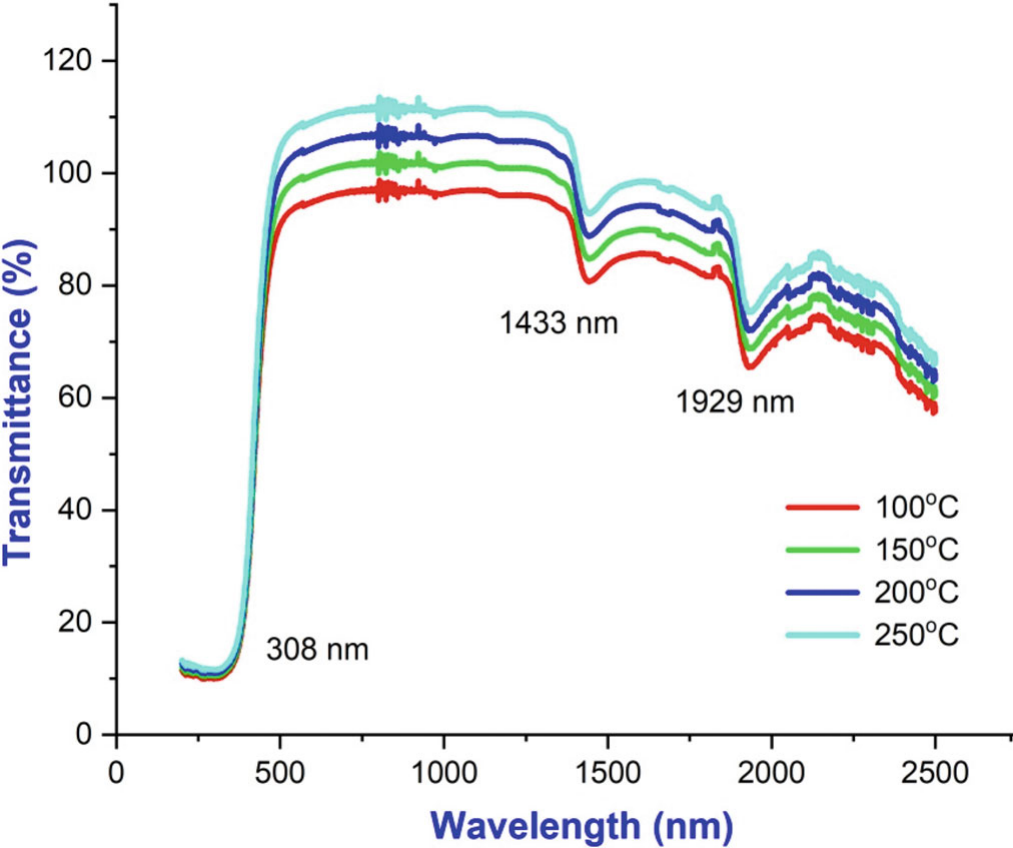
Optical transmittance spectrum of BaTiO_3_ nanoparticle at various temperatures.

BaTiO_3_ NPs synthesized are tested for their optical properties by UV–vis spectroscopy as shown in Figure 3, were performed at room temperature. In the synthesized particles, it was found that the absorbance decreased sharply with increasing wavelength, but that the absorption coefficient remained constant indicating that the size of the particles was uniform (Xu et al., 2019). An absorption peak was observed at 325 nm in the observed spectrum. As can be seen in Figure 4, BaTiO_3_ NPs were able to absorb UV light and reflect UV light. From the optical absorption spectra, the energy bandgap (*E*_g_) is calculated based on *Tauc relationships* (Sun et al., 2021). *α*hv = A(h*v*−*E*_g_)^*n*^ This equation determines the type of optical transition (*n* = 1/2 and 3/2 for direct allowed and forbidden transitions and *n* = 2 and 3 for indirect allowed and forbidden transitions) as shown in Figure 5. *A* = absorption coefficient, hv = photon energy, *e* = bandgap, *n* = constant, which determines the type of optical transition (*n* = 1/2 and 3/2 for direct allowed and forbidden transitions).

### 3.3 PL studies

**FIGURE 6:**
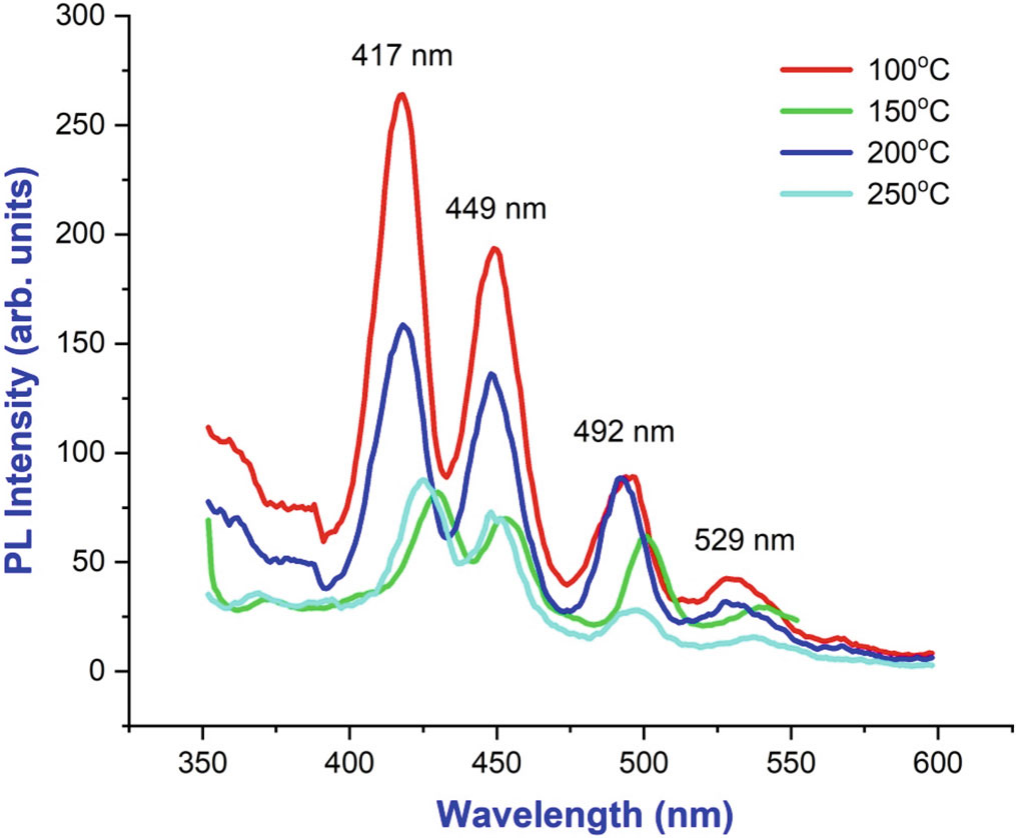
Photoluminescence spectrum of BaTiO_3_ nanoparticle at 100°C, 150°C, 200°C and 250°C.

A photoluminescence (PL) spectrometer is used to measure electronic excitement, electron configurations, defects, impurities, and the rate of recombination in semiconducting nanomaterials. It can also detect quanta of electron fluorescence. A PL spectrum was observed at room temperature for BaTiO_3_ NPs with an excitation wavelength of 350 nm Figure 6 for the compound in consideration. Both ultraviolet and visible emission regions were connected by the spectral curve between 350 and 550 nm. An electron–hole (e^−^/h^+^) pair recombination is the cause of the weak spectrum observed at the initial stage of the plot in the UV limits. Additionally, the strong spectrum observed in the plot near the visible limits was due to the presence of O_2_ vacancies and Ti interstitials. Photocatalytic studies and UV–vis studies also confirm the results from PL measurements.

### 3.4 FESEM and EDX analysis

**FIGURE 7:**
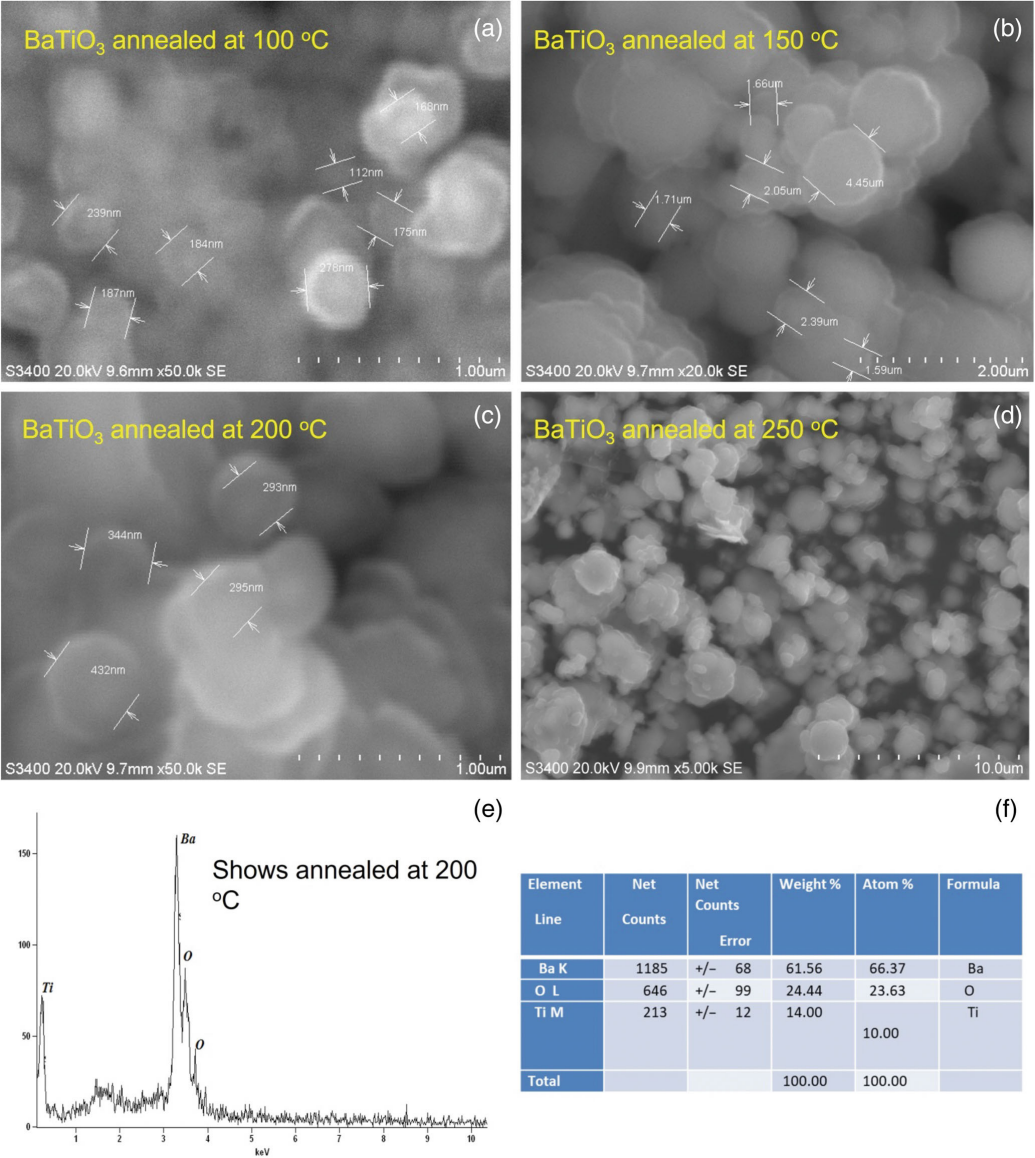
(a–f) Field emission scanning electron microscopy and EDAX images of BaTiO_3_ nanoparticle at various temperatures.

A morphological analysis of Figure 7 was conducted using FESEM micrographs of BaTiO_3_ and NPs prepared by soft chemical root‐modified solvothermal method. FESEM images Figure 7a–e show that the BaTiO_3_ nanocrystals exhibit a circular and noncircular flakes like morphology with a thickness of 3.3 nm –11.9 nm and a surface are about few 100 nm. Many of the nanoflakes have circular or spheroid unshaped, and a remaining of them are irregular surface morphology and flakes like morphology could be being proved by stacking them into innovative structure using heat treatment at different temperature range that are under studies (Liu et al., 2020). An energy dispersive x‐ray spectroscopy (EDX) technique was also utilized to determine the elemental composition of BaTiO_3_ nanoflakes synthesized as shown in Figure 7f presents EDX results graphically. Based on the EDX spectrum, it is apparent that barium, titanium, and oxygen dominate the peak, showing that these three elements were present in the synthesized NPs (Yu et al., 2021). As seen in Figure 7e, the EDX spectrum is accompanied by a graphical representation of the result of the BaTiO_3_ NPs, furthermore, and no other impurity peak dominates the peak. In addition to the verification of the presence of elemental Ti in the sample, this analysis indicates that the synthesized materials were in pure forms in Figure 7b. In this case, the sharp peak at 3.256 keV corresponded to the presence of elemental Ba.

### 3.5 Photocatalytic studies

**FIGURE 8:**
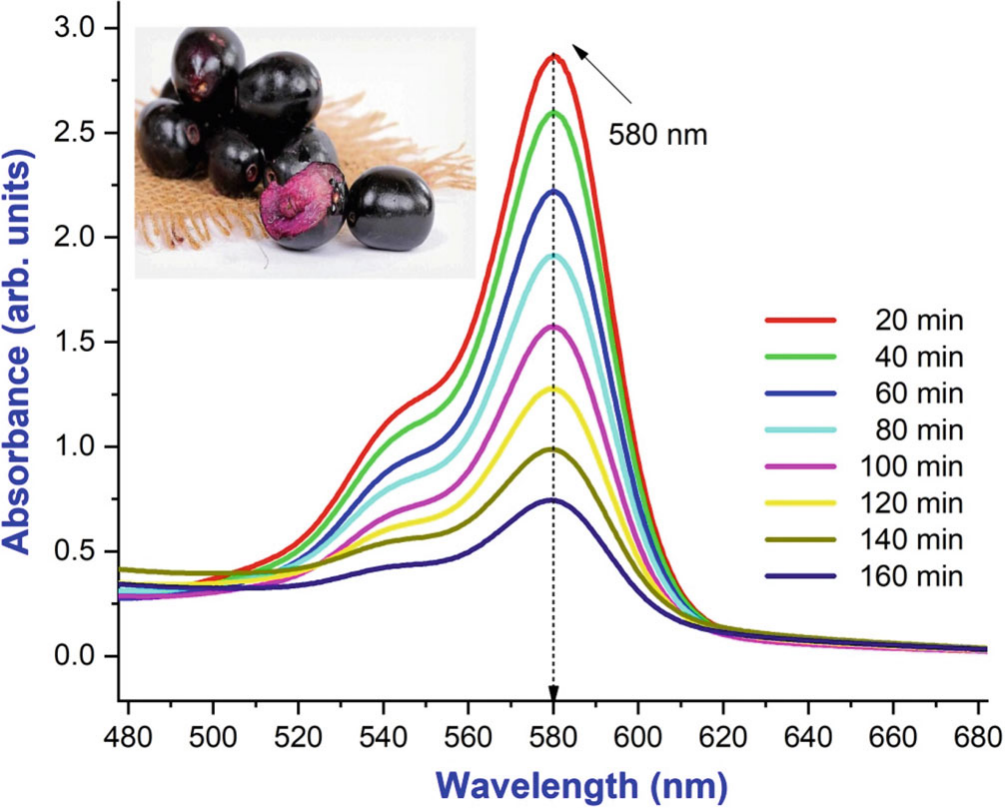
Photocatalytic natural degradation dye of novel fruit extract degradation of BaTiO_3_ at 200°C under UV light irradiation.

**FIGURE 9:**
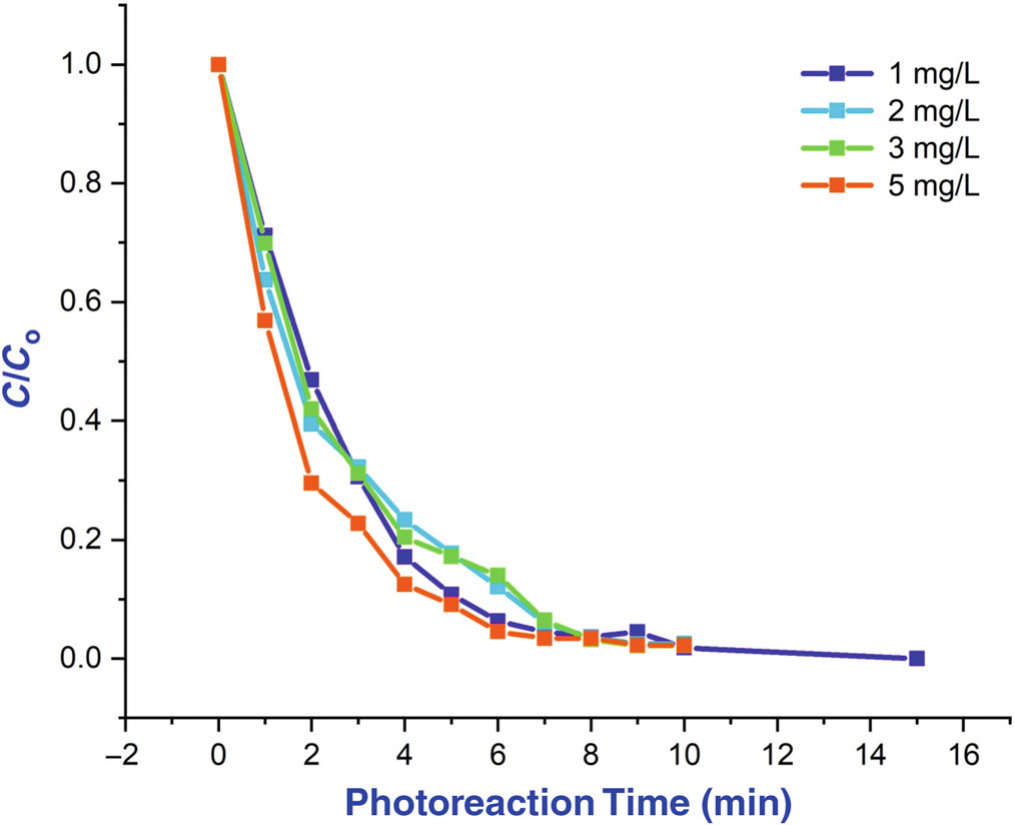
Graph of *C*/*C*_o_ versus photoreaction time of novel fruit dye by the BaTiO_3_ nanoparticle at. 1, 2, 3, and 5 mg/L under UV light irradiation.

In visible light illumination, BaTiO_3_ nanoflakes were tested for their effectiveness in degrading organic dyes. Many types of research have reported the photodegradation effect on organic dye using oxide nanomaterials in the process of neglecting unwanted foreign entities. In these aspects, BaTiO_3_ NPs behave as an effective catalytic agent in the degradation of dye from its original color, which reveals the reference those nanocatalytic particles having the ability to act as the vital candidate of industrial agent because of its rapid and efficient rate of catalytic activity (Yang et al., 2022). Therefore, the present study that the photodegradation process of synthesized BaTiO_3_ NPs was studied by the decolourization mechanism applying to organic dyes from the novel fruit extract under radiating of photons in the range of UV region at periodic space of a quarter‐hour. Preliminary conditions were set up in the spectrophotometer for observation such as optimum energy, wavelength range, well‐dissolved sample, and zero light environments. In this photocatalytic activity, the organic dye was examined to ratio the absorbance versus the wavelength of UV in the presence of barium titanium oxide in minimum concentration to enhance the decolourization reaction without affecting the nature of the sample itself. From this activity, it is observed that the expected significant maximum absorption was recorded at 580 nm as shown in the Figure 8 of the plot. At the initial time, there was no spectrum to be appeared because of the solution under buffering to specific seconds for making interaction with molecules of the dye. The buffering time depends upon the crystallite size, morphology, and dye concentration. As a result of the surface‐to‐volume (s/v) ratio nanoflakes and sustaining the deliverance of charge carriers in the presence of more oxygen vacancies and significant defects on the surface, photodegradation activity had been taking place, which is inferred in the Figure 9. Invisible light illumination was used to study the effectiveness of BaTiO_3_ nanoflakes in degrading organic dyes. Increasing the annealing temperature of the sample improved the photocatalytic activity. As shown in the recorded spectrum, each quarter‐time interval saw an exponential decrease in absorption spectral intensity. BaTiO_3_ nanoflakes are a promising candidate for photocatalysts for organic dye degradation based on the decrease in phyto‐dye molar concentration under UV radiation Figure 9. Using a UV–visible spectrophotometer, a 10‐min centrifugation at 3500 rpm was performed on the prepared aqua sample. An observed decrease in absorption characteristics at 580 nm is evidence of phyto‐dye photodegradation. As percent of photo degradation represents the concentration of organic dye solution at time 0 sec and time *t* respectively, the equation percent of photo degradation = *C*_o_−*C*/*C* × 100 was used to calculate the percentage degradation.

**FIGURE 10:**
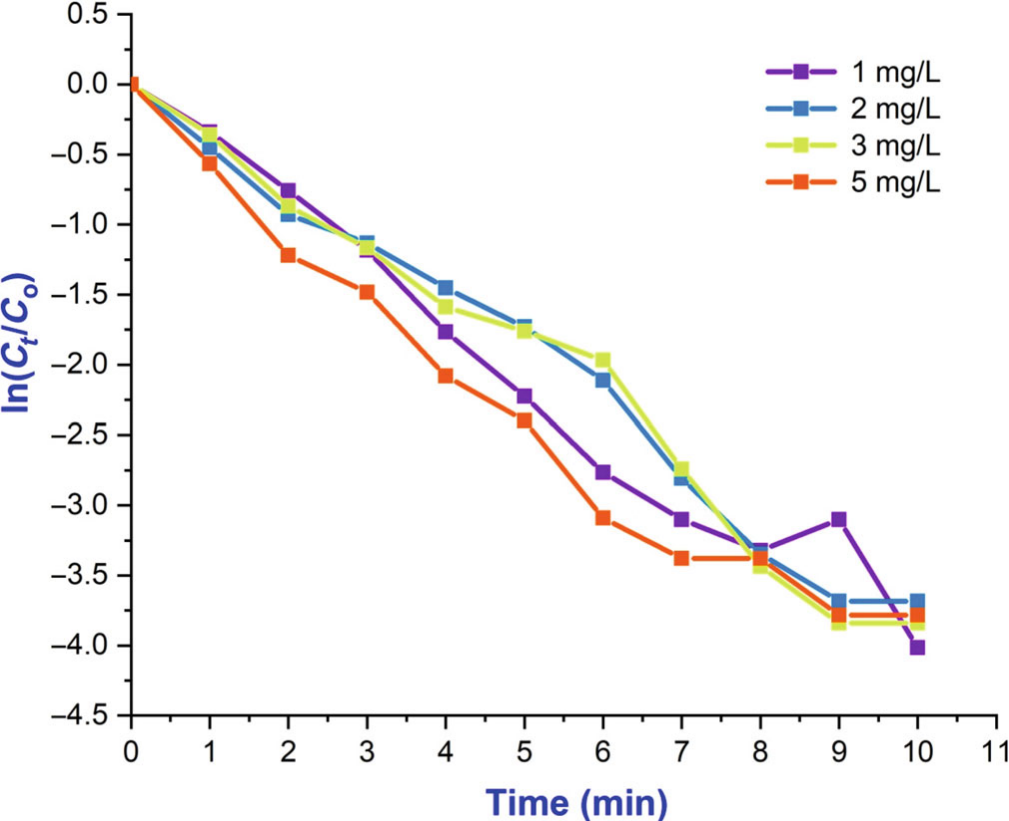
Efficiency kinetic (ln *C*_*t*_/*C*_o_ vs. time) plots of the degradation of BaTiO_3_ nanoparticle at 1, 2, 3, and 5 under UV light irradiation.

**FIGURE 11:**
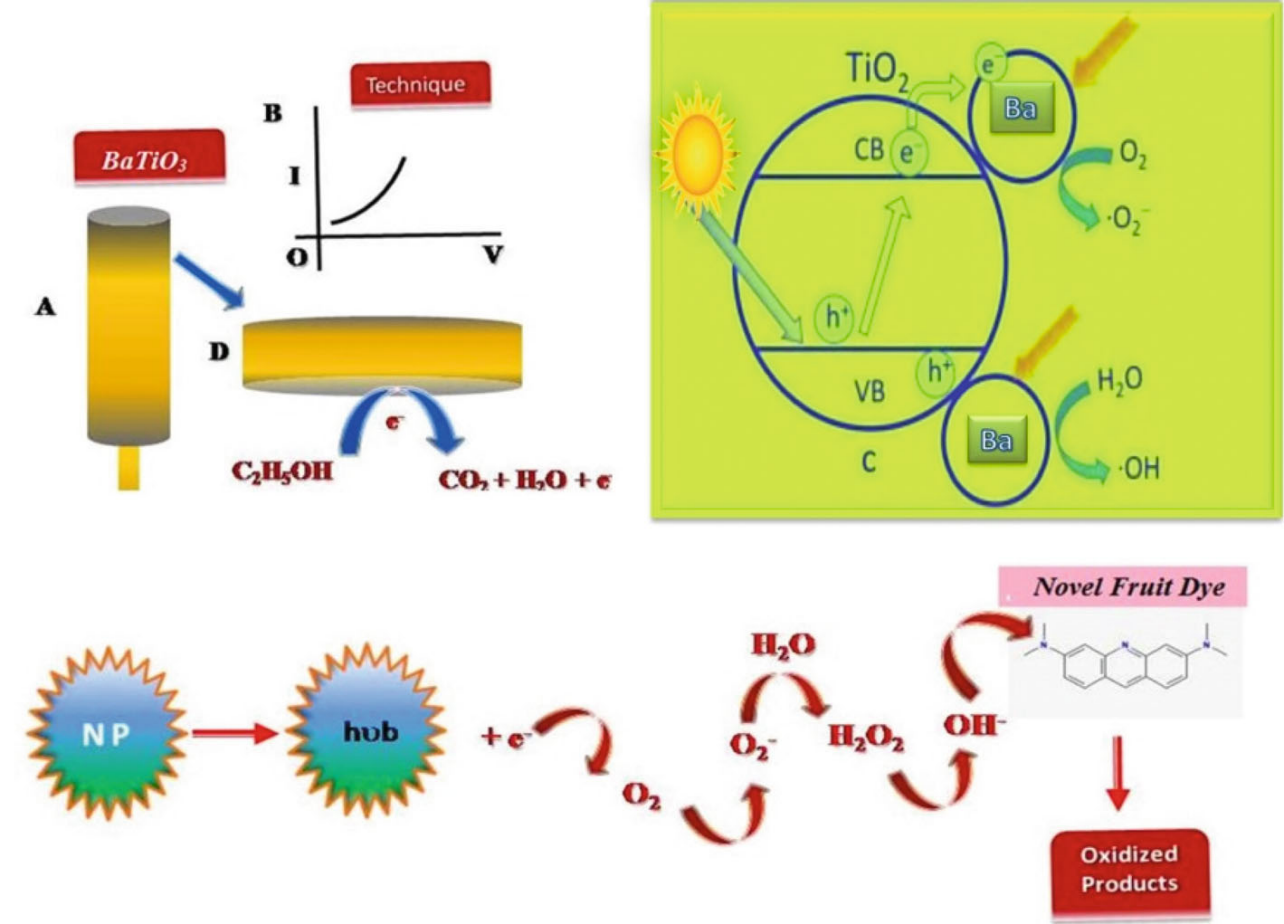
Mechanism of O_2_ and H_2_ evolution half reaction of H_2_O splitting with compatible reagents.

**FIGURE 12:**
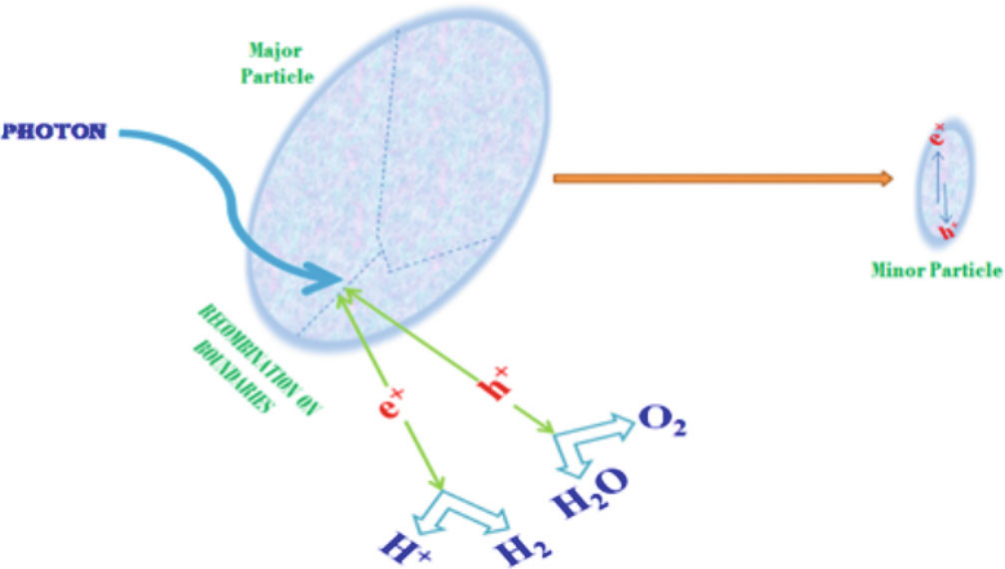
Impact of crystalline boundary and size in the photocatalytic activity.

As shown in Figure 10 Obtaining more insight into the photocatalytic activity of BaTiO_3_ nanoflakes was achieved through the kinetic study. Photocatalytic reaction rate constant = ln*C*/*C*_o_ = *kt*, is given by the pseudo‐first order law. “*k*” is reaction rate constant, while “*t*” is time between irradiation and reaction. Based on the plot in Figure 11, it appears that BaTiO_3_ nanoflakes degrade phyto‐fruit dye according to pseudo‐first order kinetics based on −ln(*C*/*C*_o_), which is a function of irradiation time. The half‐life period of a first order reaction involves 65% of the reaction's completion within a certain amount of time, where *k* represents the rate constant of decolourization (*k*). This photocatalytic activity always depends upon the surface, size, and defects of the crystallites. The maximum number of defects with significant crystallinity increases the recombination zones for photogenerated h^+^ s and photogenerated e^−^ s as shown in Figure 12. On the other hand, if the crystallite size with short boundaries of surface, decreases the photocatalytic reaction by bringing both photogenerated h^+^ and e^−^ to the active region of the half‐reaction (Huang et al., 2019).

**FIGURE 13:**
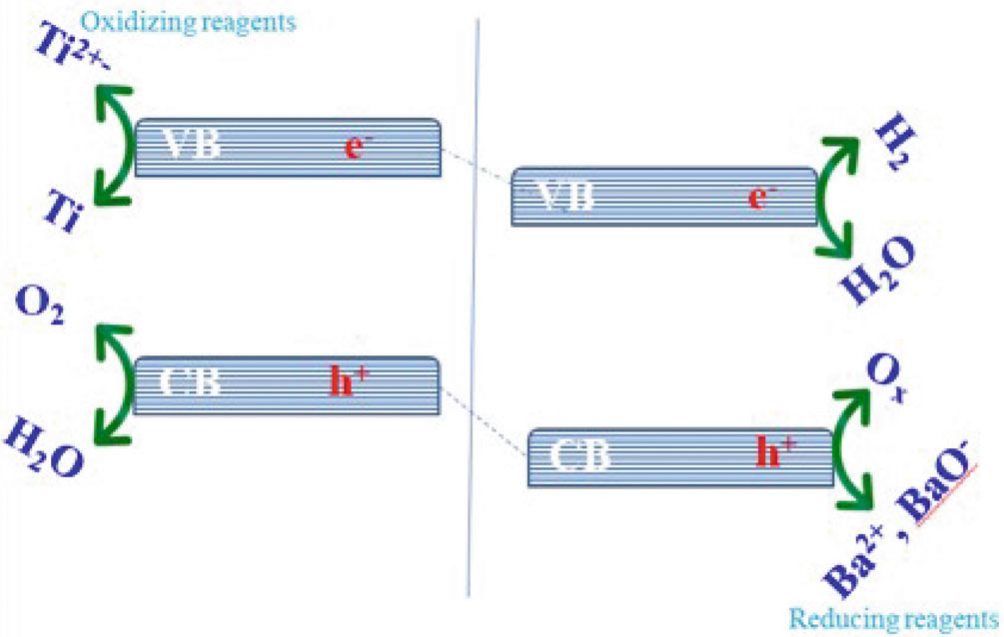
Mechanism of O_2_ and H_2_ evolution half reaction of H_2_O splitting with compatible reagents.

The Figure 13 shows that the compatible candidates of the elemental composition were utilized to gauge the PCA for H_2_O separation because of overall H_2_O splitting could be a powerful reaction (Zou et al., 2022). The important particles such as negative charge (e^−^) emitter and positive charge (h^+^) collector, which exist in the solution took part during the photodegradation activity as well as photogeneration where the holes oxidizing the fruit extract solution permanently donating the electrons by means of oxidization rather than the H_2_O. In the mechanism of water splitting process, the oxygen evolution takes place in the valence band (VB) hydrogen evolution takes place in the conduction band (CB) by utilizing the phytochemical, e^−^ consumers like Ti^2+^ and e^−ns^ emitter (Zhang et al., 2021).

## 4 CONCLUSION

In the summary of studies, a novel tetragonal BaTiO_3_ nanoflake was successfully prepared viasoft chemical root‐modified solvothermal synthesis combo method. In FESEM, it was found that the temperature variant did not significantly influence the orientation of the crystalline peak, despite XRD patterns showing circular and noncircular flakes or discs like morphology with at least a thickness of 7.625 nm and a surface area of micrometers along with some agglomeration. EDX spectra were used to confirm the element proportions in BaTiO_3_ nanoflakes. BaTiO_3_ nanocomposite displays unique photocatalytic activity when exposed to natural solar light. BaTiO_3_ is known to have the lowest optical bandgap value according to the study. In all compositions, BaTiO_3_ has the largest size, which is why its bandgap is so low. The incorporation of BaTiO_3_ nanoflakes influenced the generation of free radical's properties bringing an increase of photocatalytic, simultaneously, did make the significant variation dye concentration of the organic dye that presented a high catalytic activity.

According to our findings, 89.71% of the natural syzygium cumin is degraded by photocatalysis. By reducing electron–hole reunion by varying the temperature, BaTiO_3_ nanocomposite exhibits improved photocatalytic activity. As a plausible mechanism for the destruction of natural dyes under solar light, photocatalytic destruction has been proposed. The reaction between these reactive free radical species leads to high efficiency photodegradation with a short decay time. In addition to water treatment and environmental cleaning applications, the excellent performance of this photocatalyst makes it a promising candidate for other applications. Hence, the synthesized BaTiO_3_ nanoflakes showcase a highly significant advancement toward the development of a textiles dye recycling method.

## AUTHOR CONTRIBUTIONS

**N. N. Shafeera:** Conceptualization; methodology; software; data curation; writing – original draft; investigation; writing – review and editing. **D. Saravanakkumar:** Investigation; conceptualization; methodology; formal analysis; data curation; validation. **K. Mohamed Rafi:** Funding acquisition; investigation; validation; visualization; formal analysis; data curation. **A. Ayeshamariam:** Investigation; funding acquisition; visualization; validation; writing – review and editing; project administration; supervision; resources. **K. Kaviyarasu:** Funding acquisition; writing – review and editing; project administration; supervision; validation; visualization.

## Data Availability

The data that support the findings of this study are available from the corresponding author upon reasonable request.
